# Management of type 2 diabetes mellitus: Adherence challenges in environments of low socio-economic status

**DOI:** 10.4102/phcfm.v6i1.713

**Published:** 2014-11-06

**Authors:** Tania Steyl, Julie Phillips

**Affiliations:** 1 Department of Physiotherapy, University of the Western Cape, South Africa

## Abstract

**Background:**

The efficacy of treatment for clients with diabetes is highly dependent on the individual's ability to manage the disease. Several constraints, such as poverty, illiteracy and insufficient resources (finances and specialised healthcare professionals), especially communities of low socio-economic status, could influence clients’ ability to manage their disease.

**Aim:**

The main aim of this study was to outline the obstacles encountered by individuals with type 2 diabetes mellitus from an urban community with regard to management of their disease.

**Setting:**

The study was conducted at a primary health care facility in the Western Cape, South Africa.

**Methods:**

Ethical clearance was obtained from all relevant authorities. Eight (8) conveniently selected clients with type 2 diabetes mellitus per participating community healthcare centre (six approved centres in total) were invited to take part in focus group discussions. Twenty six clients, 15 females and 11 males, with a mean age of 58.92 years (SD = 7.33), agreed to participate. Audiotaped data were transcribed verbatim followed by content analysis and identification of themes.

**Results:**

Themes that emerged were challenges with: a healthy eating plan, physical activity, financial constraints, other people's understanding of the disease, and service received at the community healthcare centre. Verbatim quotes were used to exemplify the themes.

**Conclusion:**

Clients with type 2 diabetes mellitus experienced several challenges in the management of their disease. These challenges should be addressed to assist with better glycaemic control and to curb the emergence of diabetic complications and their attendant cost implications.

## Abstrait

**Gestion des diabétiques de type 2: les défis d'adhérence dans des environnements à faible socio-économiques**.

**Contexte:**

L'efficacité du traitement pour les clients atteints de diabète est très dépendante de la capacité de l'individu à gérer la maladie. Plusieurs contraintes telles que la pauvreté, l'analphabétisme et l'insuffisance des ressources (finances et les professionnels spécialisés de soins de santé), en particulier dans les groupes à faible socio-économique pourraient influencer la capacité des clients à gérer leur maladie.

**Objectif:**

L'objectif principal de cette étude était de décrire les obstacles rencontrés par les personnes atteintes de diabète de type 2 à partir d'une communauté urbaine en ce qui concerne la gestion de leur maladie.

**Définition:**

L’étude a été menée dans un établissement de soins de santé primaires dans le Western Cape, en Afrique du Sud.

**Méthodes:**

jeuéthique a été obtenue à partir de toutes les autorités compétentes. Huit (8) clients modèles ont été sélectionnés avec le diabète de type 2 par participant Centre de santé communautaire (six CSC approuvés au total) ont été invités à prendre part à des discussions de groupe de discussion. Vingt-six (26) clients, 15 femmes et 11 hommes avec un âge moyen de 58 à 92ans (SD = 7.33), ont accepté de participer. Les données enregistrées sur bande audio ont été transcrites intégralement suivies par l'analyse du contenu et l'identification des thèmes.

**Résultats:**

Les thèmes émergents sont les défis avec: un plan d'alimentation saine, l'activité physique, les contraintes financières, la compréhension par les autres personnes de la maladie et le service reçu au CSC. Des citations textuelles ont été utilisées pour illustrer les thèmes.

**Conclusion:**

Les clients avec diabète de type 2 ont connu plusieurs difficultés dans la gestion de leur maladie. Ces défis doivent être relevés pour aider à un meilleur contrôle de la glycémie et à réduire l'apparition de complications diabétiques et les répercussions sur les coûts de celles-ci.

## Introduction

### Key focus

The benefits of good glycaemic control in patients with diabetes mellitus (DM) have been highlighted in research, as glycaemic control is directly related to development of diabetic complications.^1^ The efficacy of treatment for clients with DM is highly dependent on the individual's ability to manage the disease.^2^ Several constraints, such as poverty, illiteracy and inadequate resources (finances and specialised healthcare professionals), especially in communities of low socio-economic status, could influence clients’ ability to manage their disease.

### Background

DM, an international pandemic, is a growing public health concern, and its prevalence is escalating exponentially with a high frequency of morbidity, premature mortality, disability, loss of productivity^3^ and socio-economic challenges.^4^ Diabetes is a chronic disease with no cure to date, and fewer than 50% of patients with type 2 DM globally meet glycaemic targets, even in developed countries.^5^ More disturbingly, less than 10% achieve blood pressure, cholesterol and glycaemic targets, even with multifactorial interventions.^6^

Despite improvement in the management of diabetes in the Western Cape, South Africa, over the past decade, a recent audit revealed that only 48% of patients with DM had an HbA1c test done in the previous year, whilst a mere 35% reached glycaemic control (HbA1c < 7%).^1^ This is of great concern as good glycaemic control is the foundation of the prevention of diabetic complications.^5^

Globally the cost implications of the management of DM are exorbitant. Almost 15% of national budgets are spent on the diagnosis and treatment of people with DM in developed countries.^7^ The International Diabetes Federation agreed that given its chronic nature, the working adults mostly affected and the resources required to manage the disease, DM is a very costly disease.^8^ Financial constraints of affected individuals as well as the healthcare sector could have a negative impact on management of the disease. Clients could not afford transport costs for clinic visits and prescribed healthy food, which was shown to negatively influence the outcome of disease management.^9^

The main focus of short-term management should be on keeping blood glucose levels in a specific range, therefore preventing hypoglycaemia or hyperglycaemia as well as long-term complications due to chronic hyperglycaemia.^10^ The long-term aims focus on avoiding hyperglycaemia as well as later complications.^11^ The benefits of good glycaemic control were recorded by two major studies, the Diabetic Control and Complication Trial conducted in the United States of America and the United Kingdom Prospective Diabetes study completed in 2003. Both concluded that glycaemic control is directly related to the development of diabetic complications.^5, 12^

The Society for Endocrinology, Metabolism and Diabetes of South Africa developed guidelines for the management of patients with diabetes in South Africa, based on the findings of international studies.^5^ Specific guidelines for diagnosis and management of type 2 DM for primary health care (PHC) were developed in 2012.^5^ These guidelines emphasise the importance of patient education to improve self-management of the disease. Knowledge about the disease as well as management skills is not enough. Effective management of chronic diseases like diabetes calls for an integrated approach with the patient, family and community taking an active supportive role.^13^ This is echoed by research which proposed that diabetes education in South Africa should involve people with DM, families, healthcare staff and communities, and should be considered as an integral and significantly important component of DM treatment and management.^14^ If DM care is to reach the ‘grassroots’, then care needs to be focused at PHC level, along with efficient administration and education of PHC staff. As DeCoster and George^15^ state: ‘Integrating diabetes self-care into daily living demands more than knowledge acquisition and skill mastery; it requires acceptance of the disease, motivation for change, instrumental and emotional support, general problem-solving skills, and control over one's life – empowerment’.

The persistence of inadequate glycaemic control could be attributed to both patients and healthcare providers. Several studies have shown that a key element in the effectiveness of health care is the effective partnership between patients and healthcare providers.^16, 17, 18^ Non-adherence, a characteristic of poor patient self-management, could increase mortality and disability as well as healthcare costs.^19^ In addition, patients’ attitudes and beliefs, knowledge of the disease, financial resources as well as social and family support have been identified as influencing adherence to management protocols.^16, 20^

Social and family support has been shown to be either a barrier or a supporting factor to adherence to a treatment and management programme as well as glycaemic control. Stress, depression, low perceptions of self-efficacy, low levels of family and social support are consistently related to low levels of diabetes self-management.^21^ In contrast, Wing and associates^22^ reported that participation of spouses in an education programme had a negative effect for obese men with type 2 DM, whilst obese women with type 2 DM adhered to the programme and achieved better results.

### Aim

Many factors contributing to adherence to care and management revolve around the patients themselves.^15^ Against this background, the main purpose of this article is to outline obstacles encountered by individuals with DM from an urban community in the Western Cape, South Africa, in management of their disease.

### Contribution to field

If the contextual factors contributing to non-adherence to prescribed treatment and management protocols for DM are unearthed, they should be kept in mind when appropriate health promotion interventions are developed and implemented to mitigate the impact of the disease. This could assist to control the costs of DM management and complications as well as improve patients’ experience regarding management of DM in South Africa.

## Research methods and design

### Study design

The study incorporated a cross-sectional qualitative design.

### Setting

The study was conducted in the Cape Metropolitan District of the Western Cape, one of the five district municipalities of the City of Cape Town, Western Cape. The Cape Metropolitan District covers an area of 2460 km^2^, has a population of 3 740 026 million people,^23^ and is divided into four substructures with 39 community health centres (CHCs), of which 22 provide diabetes care and management. Of the individuals diagnosed with type 2 DM in the Western Cape, 55% are from the Cape Metropolitan District.

### Study population and sampling strategy

Eight conveniently selected clients with type 2 DM per participating CHC (6 approved CHCs in total) were invited to take part in the focus group discussions (FGDs). Of the total of 48 invited clients, 26 agreed to participate, resulting in a response rate of 54.2%. The decision was made to conduct six FGDs, and if saturation was not reached more participants would be recruited. A total of six FGDs was conducted.

### Data collection

The FGDs were conducted at a venue organised by the respective CHCs on a day convenient for the consenting clients. The procedure was explained to the participants and each consenting participant completed an FGD binding form. Participants were asked to reflect on the challenges they experience in the management of their disease. They were encouraged to talk freely and participate fully in the discussions. The facilitator guided the discussions to permit and encourage participation from everyone in the language used by the majority of the participants (English). The decision about language medium was made by the group. The sessions were concluded when each participant said they could not think of anything else to add. Each session lasted between 45 minutes to an hour. The responses were audiotaped and recorded on paper.

### Data analysis

Data from the audiotape recordings was transcribed verbatim by an independent person with experience in transcription to produce a manuscript. A comparison was made between notes taken during the discussions to verify accuracy. Content analysis was done by extracting meaningful ideas from the participants’ opinions (coding into themes). Thereafter the transcripts were read several times by the authors to look for emerging themes. The themes identified by the authors were compared to the themes identified by an independent researcher. Grouping of the themes into broader categories was done in order to fit small categories together.

### Data storage

All tapes were destroyed once they had been transcribed and documented according to themes. The transcribed data were stored in a locked and safe place.

### Ethical considerations

Ethical approval was obtained from the relevant university authority of the University of the Western Cape, South Africa (clearance number 11/4/2), the Western Cape Department of Health (six of the seven randomly selected CHCs) and the facility managers of the six approved CHCs.

The purpose of the study was clearly explained to all consenting participants using an information sheet. Signed, written informed consent was obtained from 26 of the clients with DM prior to the FGDs. The consent form, information sheet and questionnaire were available in English, Afrikaans and isiXhosa. Participation in the study was voluntary. The participants were informed of their right to withdraw from the study at any time without any consequences. Identification codes using numbers were employed on data forms to ensure anonymity. Only the researcher and research assistant had the details of the participants. Information obtained from the informal discussions was handled with confidentiality. Participants in the discussion signed an FGD binding form in which they undertook not to disclose any information from the discussion.

Minimal perceived risks were expected in the study. However, if participants were affected by the study and experienced questions as traumatic, they would be referred to a counsellor for management. Although the participants will not directly benefit from the study, the results could be used to develop and implement appropriate health promotion strategies at PHC settings in South Africa.

### Trustworthiness

Trustworthiness of qualitative data is measured by its credibility, which in qualitative research is determined by the match between assembled realisms of the participants and the data drawn from the participants presented by the researcher.^24^ Several steps were considered to build credibility: prolonged engagement and persistent observation; member checks by giving feedback of the data to participants so that they could comment on accuracy of the recordings; responses were transcribed verbatim; and independent researchers were asked to read through the transcripts and generate the themes.

## Results

Six FGDs were facilitated by the researcher and research assistant. Twenty six clients, 15 females and 11 males, with a mean age of 58.92 years (SD = 7.33) agreed to participate.

The emerging themes generated from the thematic analysis of the FGDs were as follows, and are outlined below:

Challenges with a healthy eating plan; Challenges with physical exercise; Financial constraints; Other people's understanding of the disease; and Service received at the CHC.

Verbatim quotes are used to further exemplify the above mentioned themes with regard to challenges experienced in self-management.

### Challenges with a healthy eating plan

The majority of participants confided that they found it very difficult to follow a healthy eating plan. Their living circumstances were amongst the reasons mentioned for this:

‘My wife passed away … I stay on my own and don't cook for myself. It is so easy to just eat bread with something on it.’ (Male, 68 years)

‘I stay with my daughter and her family. I must eat whatever she cooks.’ (Female, 60 years)

‘I stay with my sister and her husband. She cooks all the food he loves. It is usually unhealthy … He likes fried foods. I don't have a choice; I must eat what I get.’ (Female, 66 years)

‘I can't cook … I just eat whatever is in the house … usually bread and things like pies.’ (Male, 57 years)

Working conditions also make it very difficult for some of the participants to follow a healthy meal plan:

‘I work on a building site. We don't have regular breaks and I am divorced. So I have to pack my own lunch and snacks. It is so much easier just to buy a pie and chips at the shop.’ (Male, 57 years)

‘To say the least, my boss is very strict and does not allow you to eat whilst you work. I work in a factory. Even though I have a letter from the hospital to say that I must eat regular small meals, he insists that I “clock in and out”. Now I lose pay!’ (Female, 56 years)

### Challenges with physical exercise

The clients expressed awareness of the importance of physical activity, although some are not engaging in physical activity. Safety in their neighbourhoods was also of great concern and expressed by the majority of participants in the FGDs. The following quotations illustrate their sentiments:

‘I wish there was a place in the community where I could go to exercise … like the church hall or so. And I don't feel safe to exercise around the house.’ (Female, 63 years)

‘I get up early in the morning to go to work and then I get back very late. It is already dark outside. It is really too dangerous to go for a walk then.’ (Male, 60 years)

Participants also stated that lack of time hampers their participation in physical activity. This is reflected in the statements below:

‘I work full-time in an office and when I get home I must do all my housework myself and cook for the family. I really don't have time to exercise during the week.’ (Female, 47 years)

‘I work half-day and then look after my grandchildren in the afternoon. There is no time to do formal exercise.’ (Female, 52 years)

Changing a bad habit was also reported to be a challenge when it comes to exercise:

‘I am very lazy … I don't even like to walk, I actually hate any form of exercise.’ (Male, 69 years)

‘I am so overweight all the years. I don't have the energy to exercise. I only do some of my housework. Otherwise I sit all day and watch TV.’ (Female, 63 years)

There was a sentiment amongst participants that their general feeling of well-being as well as physical disabilities precluded them from exercising:

‘Some days I am too tired to get out of bed. I also have a heart problem.’ (Female, 68 years)

‘My foot was amputated last year and now I must walk with crutches. My hands get sore and tired when I walk too much.’ (Male, 64 years)

### Financial constraints

Lack of money was reported to be a key challenge in the management of type 2 DM, as it causes difficulty for the participants in buying healthy food and going to the CHCs for regular check-ups. This is illustrated in the following excerpts:

‘I get a grant. My two grandchildren stay with us. We don't have money to buy all the right foods like the stuff that you can use in the place of sugar and all the vegetables. The children get a plate of food at school and sometimes we just eat dry bread for days.’ (Female, 51 years)

‘I only have R450 in a month for groceries. So I buy cheap stuff, not the vegetables that are very expensive.’ (Female, 59 years)

‘My daughter takes my grant ... so I can't buy food myself … I have no money.’ (Female, 66 years)

‘Healthy food is a lot of money ... actually, all foods are expensive. Pap and bread are the cheapest.’ (Female, 71 years)

‘I must take a taxi to go to the clinic every month. It is too far to walk and I sometimes feel too weak to walk. It is very expensive. I only get a grant and have to pay a lot for rent.’ (Male, 68 years)

‘My neighbour charges me a lot to take me to the day hospital and fetch me again. I can't get to the taxi rank; it is too far from my house.’ (Female, 63 years)

### Other people's understanding of the disease

A few of the participants mentioned that not all people understand what the disease entails. It causes them to struggle with proper self-management of the disease:

‘It is very difficult at parties or family functions. They [*the family*] look at you in a funny way if you don't eat the food like pies and pastries. So now I just eat it, although I know it will badly affect my sugar. I am tired of explaining why I should not have it.’ (Female, 47 years)

‘I can't help that my clinic appointment is sometimes on the day of deadlines at the work. My boss refuses then that I can go for my appointment. Then I don't always have medication.’ (Male, 60 years)

### Service received at the CHC

The majority of the participants complained about the service they received at the CHC. The long waiting times to see the doctor or sister and to get their medication at the pharmacy are a major problem:

‘The long queue at the Pharmacy is very bad. I sometimes wait half a day before I get my medication. And then it is not always the right tablets! I also once did not get enough medication to last me until my next visit.’ (Female, 54 years)

‘The staff is always in a hurry; so they don't have a lot of time to spend with you. I don't get all the information I want as they must help the other people that are waiting outside.’ (Male, 68 years)

‘I sometimes leave without seeing the doctor or sister, because it just gets too much to wait more than three hours in the queue! This also caused me not go to the clinic a few times for my appointments.’ (Female, 47 years)

‘There is not enough staff. They just do the finger-prick test and take your blood pressure, but they don't tell you what it is. If you don't ask them, you will never know if your sugar and blood pressure is high or not. But it is the Government's fault. They should employ more people to work at the clinics.’ (Female, 51 years)

## Discussion

### Outline of the results

The importance of disinterring the perceived circumstantial factors contributing to non-adherence to self-management strategies in clients with DM has been highlighted. This study aimed to assess the challenges experienced by clients with DM in the management of their disease at PHC settings in the Western Cape, South Africa. Recognising the challenges that clients experience in the management of their disease is crucial for development and implementation of health promotion strategies.

Certain challenges with physical exercise were communicated by the participants, barriers to which included safety in the neighbourhood, lack of time, changing a bad habit, and a general feeling of well-being or health-related conditions. These findings replicate those in previous studies that examined exercise preferences and barriers to physical activity in people with type 2 DM in both the developing and developed context.^25, 26, 27, 28^ Furthermore, research reported that people's perception of unsafe neighbourhoods resulted in a broad range of negative effects, including physical inactivity^29^ that also results in obesity.^30, 31^ The participants in the current study are from a socio-economically deprived community, hence the lack of access to safe pathways or parks, high levels of crime and low sense of safety in their neighbourhood.

Apart from the environmental factors contributing to physical inactivity, personal factors such as a low motivation to change a bad habit are also a major challenge for some clients. It is very difficult to change a bad habit if you do not have the motivation or if it is not regarded as a priority.^32^ The clients’ inability to set obtainable goals for themselves and to stick to them could further aggravate their sedentary habits. It is therefore imperative that health promotion strategies for clients with DM should include ways to improve self-efficacy and to set reachable goals. Furthermore, they should take into consideration perceived obstacles to adherence.^33^

As for following a healthy eating plan, the majority of participants reported that they find it very difficult to follow a healthy eating plan. Several reasons were given, i.e. living circumstances, work environment, financial constraints and other people's understanding of the disease. These results are similar to those found in research from other developing countries.^25, 26, 31^ In the present study it was evident that the clients did not have the knowledge to substitute unhealthy food with healthier options of more or less the same price; they were of the opinion that all healthy foods are expensive.

Regarding diabetes care, a number of challenges related to the healthcare services were reported. Almost all of the participants were concerned about the shortage of staff, especially the availability of pharmacists, medical doctors and nursing staff. Some of the participants were unhappy at the short consultation times as well as seldom seeing a medical doctor. The same concerns were reported in a Ghanaian study.^34^ The long waiting time in the clinic waiting room – sometimes more than two hours – was also stated to be a problem for the clients. Some of the participants noted that this was a factor in their decision to miss their scheduled appointments, an observation consistent with those of other researchers.^35^

### Practical implications

The social environment carries both the potential to underpin poor care and management as well as to ameliorate unhealthy lifestyles and to support adoption of a healthy lifestyle and provision of adequate care and management of the disease. Identification of perceived barriers to self-management and instruction on how to overcome them could considerably enhance adherence to prescribed treatment.

### Limitations of the study

Data were analysed cross-sectionally, therefore limiting the ability to make causal inferences.

### Recommendations

Although addressing issues such as neighbourhood safety and gang violence seems like a daunting task, collaboration between several national and local government departments and community forums is required to find interim solutions. Tailored physical activity classes in community halls by lay health educators could be seen as a possible solution to increase physical activity participation in this community. This type of intervention will not only provide a safe environment for physical activity but also address social support issues, such as the desire to exercise with others and provide opportunities for socialisation. It is also in line with the *Health Care 2030* document of the Western Cape Department of Health^36^ that envisages a more prominent community-based service platform where teams will be assigned with the responsibility of the health of a distinct population. Lastly, the shortage of staff should be addressed at all PHC facilities in South Africa.

## Conclusion

The above findings emphasise the challenges that clients with DM experience that negatively influence their self-management strategies. It further points to the fact that clients are disempowered, with seemingly little control over their environment and social interactions. It calls for action to be taken by national and local health departments to empower clients with DM through development and implementation of health promotion strategies that bear the reported challenges in mind. This will assist clients with DM to adopt and maintain a healthy lifestyle.
